# A new novel mutation in *FBN1* causes autosomal dominant Marfan syndrome in a Chinese family

**Published:** 2012-01-13

**Authors:** Jiamei Dong, Juan Bu, Wei Du, Yuan Li, Yanlei Jia, Jianchang Li, Xiaoli Meng, Minghui Yuan, Xiaojuan Peng, Aimin Zhou, Lejin Wang

**Affiliations:** 1 Department of Ophthalmology, Peking University Third Hospital, Key Laboratory of Vision Loss and Restoration, Ministry of Education, Beijing, P.R. China; 2 Department of Cardiology, Party School of Central Committee of C.P.C., Beijing, P.R. China; 3 Department of Ophthalmology, Subei People's Hospital of Jiangsu Province; Clinical Medical College, Yangzhou University, Jiangsu, P.R. China; 4 Department of Ophthalmology, Jianping County Hospital, Liaoning, P.R. China; 5 Department of Ophthalmology, The Municipal Hospital of ZaoZhuang, Shandong, P. R. China; 6 Department of ophthalmology, General Hospital of the Second Artillery, Beijing, P. R. China; 7 Department of O.E.N.T, the 188 PLA hospital, Chaozhou, P. R. China; 8 Clinical Chemistry Program, Department of Chemistry, Cleveland State University,Cleveland, OH

## Abstract

**Purpose:**

Screening of mutations in the fibrillin-1 (*FBN1*) gene in a Chinese family with autosomal dominant Marfan syndrome (MFS).

**Methods:**

It has been reported that *FBN1* mutations account for approximately 90% of Autosomal Dominant MFS. *FBN1* mutations were analyzed in a Chinese family of 36 members including 13 MFS patients. The genomic DNAs from blood leukocytes of the patients and their relatives were isolated and the entire coding region of *FBN1* was amplified by PCR. The sequence of *FBN1* was dertermined with an ABI 3100 Genetic Analyzer.

**Results:**

A previously unreported the missense mutation G214S (caused by a 640 A→G heterozygous change) in *FBN1* was identified in the Chinese family. The mutation was associated with the disease phenotype in patients, but not detected in their relatives or in the 100 normal controls.

**Conclusions:**

This is the first report of molecular characterization of *FBN1* in the MFS family of Chinese origin. Our results expand the spectrum of *FBN1* mutations causing MFS and further confirm the role of *FBN1* in the pathogenesis of MFS. Direct sequencing of the mutation in *FBN1* may be used for diagnosis of MFS.

## Introduction

Marfan syndrome (MFS) is an inherited, autosomal dominant,systemic disorder of connective tissue. The estimated prevalence of MFS is one in 10,000 to 20,000 individuals. It is well known that three major systems: skeletal, ocular, and cardiovascular are affected by the disease. The clinical criteria for MFS include a combination of skeletal manifestations, ectopia lentis, dural ectasia, and dilatation or dissection of the ascending aorta.

The main pathogenesis of MFS is currently thought to be driven by mechanisms due to haploinsufficiency of wild-type fibrillin-1 [1]. Fibrillins are ubiquitous extracellular matrix molecules that assemble into microfibrils [2-4] and target growth factors to the extracellular matrix [5-7]. Fibrillin-1 mutations disrupt microfibril formation, thereby resulting in fibrillin protein abnormalities, and subsequently weakening the connective tissue. More than 1000 mutations have been identified in the gene for fibrillin-1 (*FBN1*) [8]. Most mutations are unique for a certain MFS family, and only approximately 10% of mutations are recurrent in different families [9].

*FBN1* is a 230-kb gene with 65 exons that encodes the structural protein fibrillin-1, located at chromosome 15q-21.1. In this study, we analyzed a four-generation, non-consanguineous Chinese family, which has been diagnosed as MFS, and identified a mutation in the 7th exon of *FBN1* (c. 640 A→G) resulting in the substitution of glycine by serine at codon 214 (p. G214S). Our data of *FBN1* mutations causing MFS further confirm the role of *FBN1* in the pathogenesis of MFS. Obviously this is the first report of molecular characterization in *FBN1* in the Chinese MFS family.

## Methods

Clinical data and 5 ml of blood samples were collected from a Chinese Zhang family with MFS. The patient underwent complete physical and ophthalmic examinations. The Institutional Review Board approved the project and investigators followed the principles of the Declaration of Helskinki. Informed consent was obtained from each person.

To identify constitutional mutations, blood specimens (8 ml) were collected in EDTA, and genomic DNA was extracted from peripheral blood cells according to a standard protocol (Roche Diagnostics Corporation, Indianapolis, IN). Briefly, all the exons and exon-intron boundaries of *FBN1* were amplified by using the standard PCR buffer system with primers listed in Appendix 1. PCR reactions were each performed in a 10 µl volume, containing 1.5 mM MgCl_2_, 0.4 mM of each primer, 200 µM dNTPs, 1 U Taq DNA polymerase, and 10-20 ng template DNA. Amplification was performed with an initial denaturation for 3 min at 95 °C, followed by 30 cycles of denaturation at 95 °C for 1 min, annealing at 55 °C for 1 min, extension at 72 °C for 1 min, and a final extension at 72 °C for 3 min.

PCR products were purified by using a PCR product purification kit (QIAquick; Qiagen, Valencia, CA). Purified PCR products were sequenced using the BigDye Terminator Cycle Sequencing v3.1 kit (Applied Biosystems, Foster City, CA). Briefly, about 10 ng of template DNA is added to each reaction and using a temperature program which included 25 cycles of denaturation at 97 °C for 30 s, annealing at 50 °C for 15 s, and extension at 60 °C for 4 min. All samples were analyzed in an ABI Prism 310 Genetic Analyzer (Applied Biosystems). The *FBN1* CDNA reference sequence with GenBank accession number NC_000015.9 was used (National Center for Biotechnical Information [NCBI], Bethesda, Md).

## Results

**Figure 1:**
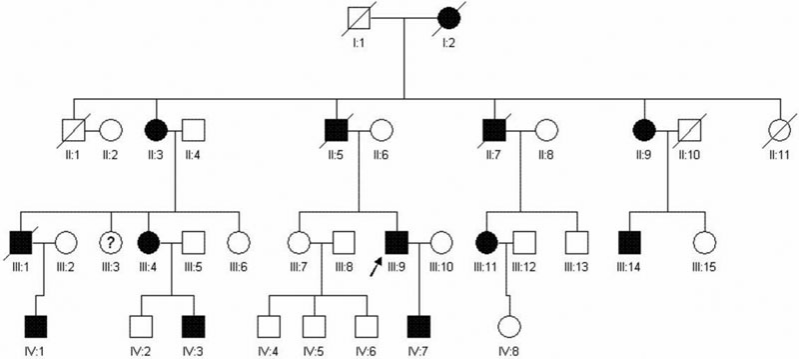
The pedigree of the family is shown. Squares and circles indicate males and females, respectively, and the darkened symbols represent the affected members. The patient above the arrow is the proband.

The patients including 8 males and 5 females were from a family in Guangdong Province, China (Figure 1). All of these patients in the family manifested various reduced visual acuities with a bilateral lens dislocation and high myopia. Aortic root dilatation was present in 2 of the 13 patients. One patient had an aortic aneurysm together with either aortic valve stenosis or aortic valve insufficiency. The other patient had a cloverleaf appearance in the cross-section, but the diameter of his aortic root was apparently normal. All patients had facial and skeletal features of MFS including joint laxity, dolichostenomelia, pectus excavatum or pectus carinatum, and scoliosis (Table 1). The cause of death for two patients was cardiovascular malformations.

**Table 1:** Clinical evaluation of affected family members.

Patient ID	II:3	II:9	II:4	II:9	II:11	II:14	IV:1	IV:3	IV:7
Age	69	59	41	37	32	26	12	9	5
Sex	F	F	F	M	F	M	M	M	M
Ocular system									
(1) Ectopia lentis	+	+	+	OEL*	+	+	+	+	+
(2) Myopia	+	+	+	OEL*	+	+	+	+	+
(3) Strabismus	+	－	－	－	－	－	+	－	－
(4) Glaucoma	－	－	－	+	－	－	－	－	－
(5) Retinal detachment	－	－	－	－	－	－	+	－	－
Cardiovascular system									
(1) Aortic root dimension (mm)	31.6	29	27.5	39.2	26	25.1	24.9	24	20.3
(2) Mitral valve prolapse	－	－	－	+	－	－	－	－	－
(3) aortic aneurysm	+	－	－	－	－	－	－	－	－
Skeletal system									
(1) Height (H;cm)	168	170	172	192	174	198	171	168	145
(2) Arm span (AS;cm)	173	175	178	201	180	208	179	177	149
(3) AS/H	1.03	1.03	1.03	1.05	1.03	1.05	1.04	1.05	1.02
(4) Scoliosis	－	－	－	+	－	－	－	－	－
(5) Arachnodactyly	+	+	+	+	+	+	+	+	－
(6) Joint hypermobility	+	+	－	－	－	－	－	－	－
(7) pectus excavatum	+	+	－	－	－	－	－	－	－
(8) Pectus carinatum	－	－	－	－	－	－	+	+	－
Other manifestations									
(1) Hyperextensible skin	+	+	－	－	－	－	－	－	－
(2) Striae	+	+	－	－	－	－	－	－	－
(3) Hernia	－	－	－	－	－	－	－	－	+

* Operated for ectopia lentis.

**Figure 2:**
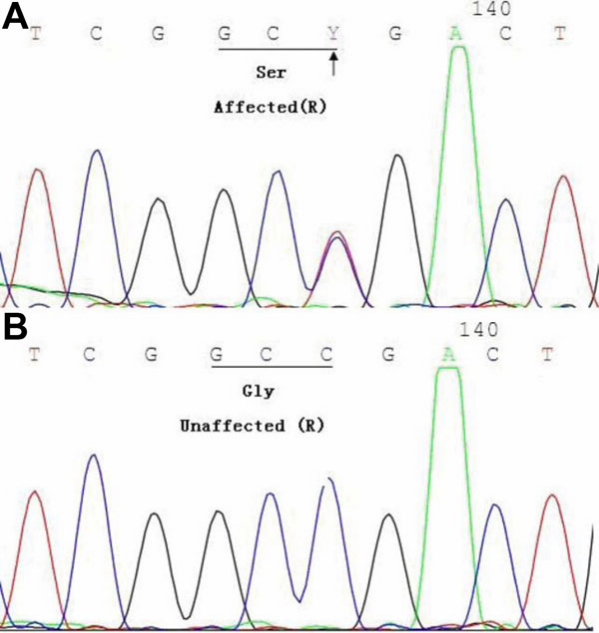
A novel *FBN1* missense mutation in extron 7. The partial nucleotide sequence of *FBN1* in is shown. **A**: The corresponding normal sequence in an unaffected family member. **B**: A heterozygous change A→G (indicated by the arrow) was identified in an affected family member.

Sequencing of the 65 coding exons of *FBN1* in the patients revealed a mutation (c. 640 A→G; Figure 2) which resulted in substitution of glycine by serine (G214S) in exon 7. The mutation was linked to the disease phenotype in all patients, but not found in other unaffected relatives or in the 100 normal controls.

## Discussion

MFS is diagnosed using the Ghent criteria, a group of clinical findings that are specific for MFS. The Ghent criteria is through a comprehensive assessment largely based on a combination of major and minor clinical manifestations in the various organ systems and the family history [10]. Although the clinical manifestations of the cardiovascular, ocular, and skeletal systems are important criteria for diagnosis of the disease, a family genetic history can also provide useful information for determining etiology and designing treatment strategy. For example, an individual with these clinical features such as tall, thin body habitus, long limbs, arachnodactyly, pectus deformities, and sometimes scoliosis with family history may be suggestive of a diagnosis of Marfan’s syndrome [11].

Cardiovascular malformations are the most life-threatening presentations of MFS. Affected patients are at risk for aortic dissection and/or severe ocular and orthopedic disorders. Although we have observed the following cardiovascular manifestations of MFS: mitral valve prolapse, calcification of the mitral valve annulus and dilatation or dissection of the descending aorta, the prevalence of cardiovascular manifestations is not very high in this family.

The clinical manifestations of the MFS patients in the family become more evident with age. The most common symptom among them is myopia and 100% of the patients have ectopia lentis. Besides, there was one patient with retinal detachment and two patients underwent IOL surgery.

The pathogenesis of the Marfan’s syndrome has not been fully elucidated. *FBN1* mutations are believed to exert a dominant negative effect [12]. In this study we identified a novel *FBN1* mutation in patients by genotype-phenotype analysis, which perfectly met the Ghent criteria (p=0.005). The mutation was also detected in those who had ectopia lentis (EL; p<0.0001) [13].

In rare cases, MFS is caused by a mutation in the transforming growth factor beta (*TGF-β*) receptor 1 or 2 genes [14-16].

The coding sequence of *FBN1* is spread over 65 coding exons. FBN1 is comprised mainly of repeated modules such as epidermal-growth-factor like (EGF) domains and transforming growth factor β1 binding protein-like (TB) domains. There are a total of 47 EGF domains on the protein. Among them, 43 of which are of the calcium-binding (cb) type (cb-EGF). There are also 7 TB domains distributed throughout FBN1, separated by variable numbers of the tandem repeated EGF domains [17-19]. Most mutations of FBN1 occur in the EGF domains [20].

FBN1 mutations disrupt microfibril formation, thereby resulting in fibrillin protein abnormalities, and subsequently weakening the connective tissue [21]. Over 601 distinct mutations have been documented in the Marfan mutation database [22]. Two-thirds of the mutations are missense mutations, and the majority of these are cysteine substitutions. Nonsense mutations account for about 10% of all reported mutations. Small insertions, deletions, or duplications represent about 13% among these mutations. Another 13% of the reported mutations are caused by various classes of splicing errors [23].

Interestingly, mutations frequently occur in exons 2, 15, 22, 27, 46, 55, and 62 but much less in exons 7, 41, and 65 [24] although the cause remains to be elucidated. According to Universal Marfan Database – FBN1 (UMD-FBN), only 2 different mutations in exon7 have been reported [25,26].

The new mutation we identified was a missense mutation in exon 7 (640 A→G heterozygous change) of *FBN1* in the Chinese family. This mutation was found in all patients, but not in unaffected family members and the 100 unrelated controls, suggesting that the new mutation may be responsible for the pathogenesis of MFS in this family.

Sequences for TB domains found in fibrillin show a high level of amino acid conservation. The TB domain is characterized by eight cysteines predicted to form four intraomolecular disulphide bonds. There are totally 11 reported mutations in the TB domains of human FBN1 which are associated with MFS. Structural analysis implicates that those mutations involving cysteines (C661R, C711Y, C996R, and C1589F) are likely to cause domain misfolding, since one of the four disulfide bonds may be disrupted. Three mutations related to either a frameshift (N1713 and E2105) or the formation of a premature stop codon (Y2113X) cause disruption of the primary sequence of this protein. Point mutations in the TB domains, including A705T, V984I, G1013R, and K1023N have also been reported [27]. Here we report that the new mutation we identified in the TB1 domain may interfere with microfibrillar assembly via producing an unstable mutant protein cleavage products.

### 

#### Conclusion

We have identified a single missense mutation in *FBN1* (c.640 A→G). in a Chinese family with Marfan syndrome. Our results expand the spectrum of *FBN1* mutations causing MFS, and further confirm the role of *FBN1* in the pathogenesis of MFS. Direct sequencing of the *FBN1* mutation could be used for diagnosis of MFS.

## Appendix 1.

Appendix 1. Primers used to amplify the exons of *FBN1*. To access the data, click or select the words “Appendix 1.” This will initiate the download of a compressed (pdf) archive that contains the file.
